# Clinical Presentation of Enterovirus D68 in a Swiss Pediatric University Center

**DOI:** 10.1097/INF.0000000000004503

**Published:** 2024-08-14

**Authors:** Chantal Ott, Gilles Dutilh, Josiane Reist, Roland Bingisser, Adrian Egli, Ulrich Heininger

**Affiliations:** * From the Applied Microbiology Research, Department of Biomedicine, University of Basel; † Department of Pediatric infectious diseases, Children University Hospital Basel; ‡ Department of Clinical Research, University of Basel; § Emergency Department, University Hospital Basel; ¶ Department of Clinical Bacteriology and Microbiology, University Hospital Basel, Basel; ∥ Institute for Medical Microbiology, University of Zurich, Zurich, Switzerland.

**Keywords:** PRES score, PCR, clinical burden, enterovirus, rhinovirus

## Abstract

**Background:**

Enterovirus D68 (EV-D68) is responsible for millions of infections. In the last decade, there has been an increase in the number of children requiring hospital or critical care admission due to severe respiratory illness. Nevertheless, the epidemiological and clinical importance of EV-D68 infections remains unclear.

**Objective:**

We aimed to determine the local prevalence of EV-D68 infection in pediatric patients and to characterize its clinical presentation and disease burden compared with non-EV-D68 enterovirus and human rhinovirus (RV) infections.

**Study design:**

We performed a retrospective single-center study of children presenting with respiratory symptoms and positive respiratory panel polymerase chain reaction for EV/RV from November 2018 to December 2019. We tested EV/RV positive specimens with an EV-D68-specific polymerase chain reaction to discriminate EV-D68, non-EV-D68 and RV and compared their respective clinical presentation, outcomes and treatment.

**Results:**

We identified 224 patients (median age 21 months), of which 16 (7%) were EV-D68 positive. They presented with cough (88%), wheezing (62%) and dyspnea (75%). EV-D68 infection had an odds ratio regarding pediatric respiratory severity-score of 11.6 relative to non-EV-D68 [confidence intervals (CI): 3.51–41.14], and of 9.9 (CI: 3.75–27.95) relative to RV. The fitted logistic regression showed that the odds of intensive care were 5 times more likely with EV-D68 than RV infection (CI: 1.32–19.28; *P* = 0.001). Patients with EV-D68 infections were more likely to receive medical support in the form of supplementary oxygen, antibiotics and steroids.

**Conclusions:**

EV-D68 infection is associated with higher morbidity and a higher likelihood of intensive care treatment than non-EV-D68 and RV infections.

Enteroviruses (EVs) are responsible for more than 10 million infections and several thousand hospital admissions every year in the United States alone.^1^ Together with human rhinoviruses (RV), these viruses are among the most common pathogens associated with infectious respiratory diseases in humans.^2,3^ EVs cause a broad spectrum of clinical phenotypes at any age, including hand-foot-mouth disease (primarily in young children), acute respiratory infections, myocarditis, neonatal sepsis and neurologic illnesses, such as aseptic meningitis, encephalitis or paralysis. Ranging from very mild to fatal infections.^4,5^

Enterovirus D68 (EV-D68) belongs to the species enterovirus D and was first isolated in 1962 from 4 children with severe respiratory tract infections (RTI) presenting with pneumonia and bronchiolitis.^1^ EV-D68 and RV are sensitive to acidic environments and prefer lower temperatures, making the nasal and respiratory mucosa more hospitable environments than the gastrointestinal tract. Therefore, they cause more respiratory symptoms than other types of enterovirus (non-EV-D68).

In 2014, a massive outbreak of EV-D68 infections caused over 1000 severe respiratory infections in the United States.^6–8^ Furthermore, at the same time an increased number of EV-D68 infections was also documented in Canada, Europe, Asia and Australia,^9^ resulting in significant public health attention. Most cases were associated with respiratory illness in young children, that is, under 4 years of age. In this cohort, many patients require hospitalization or intensive care unit (ICU) admission, and the disease is also associated with sporadic deaths.^10,11^ In addition, an increased number of EV-D68 infections were associated with severe neurological disease, mainly acute flaccid myelitis and acute flaccid paralysis (AFM and AFP).^12–14^

After the 2014 outbreak, active surveillance of infections caused by EV-D68 was implemented in many countries in Asia, Europe and America. In Switzerland, however, no surveillance system for this important pediatric infectious disease is available. Surveillance data demonstrated that EV-D68 continues to circulate globally. In many countries, particularly in Europe and North America, EV-D68 exhibited a biennial pattern, with peaks in the late summers and autumns of even-numbered years, for example, 2014, 2016 and 2018. Other countries have reported odd-year outbreaks, such as Thailand (2009/2011), Australia (2011/2013) and Japan (2015). In 2018, ongoing circulation was reported from Europe (in Wales, Italy, France), and in the United States.^15,16^ EV-D68 has mostly been reported in children, whereas it is less well-studied in adults. Also, comparative studies of disease characteristics in children versus adults are lacking.

The high number of cases with severe respiratory infections and the association with neurological complications suggest that EV-D68 is more virulent than previously thought.^17–20^ Moreover, the simultaneous circulation of similar strains of EV-D68 in different countries indicates that this virus can periodically cause global epidemics.^21^ Further studies are required to fully understand the epidemiological and clinical importance of EV-D68 infections across different age groups. A better understanding of its epidemiology, clinical presentation and outcomes may allow the development of effective preventive and therapeutic measures.

Our aims were to determine the prevalence and to characterize the clinical presentation, morbidity and outcomes (intensive care) of EV-D68 infection and other RVs and EVs in pediatric patients in Basel, Switzerland, during November 2018 to December 2019.

## METHODS/STUDY DESIGN

### Patients and Samples

This is a retrospective cohort study of patients admitted and treated at the University Children’s Hospital Basel (129 beds), a tertiary care center. We included pediatric patients from November 2018 to December 2019, as the nasopharyngeal samples from children were only routinely stored from November 2018.

We included all EV/RV polymerase chain reaction (PCR) positive isolates using the Filmarray Biofire Respiratory Panel (bioMérieux, Lyon, France; pre-COVID-19 assay design). Patients underwent respiratory viral testing as clinically indicated at the time of their evaluation, at the discretion of the treating physician. Indications for testing were symptoms of a respiratory infection in patients with the intention to be hospitalized, as well as very immunocompromised patients with very mild symptoms.

The nasal-pharyngeal swabs left-over material was collected and stored at −80 °C until further processing with specific PCR assays.

### Ethics

The study was approved by the ethical review board of North-Western Switzerland (EKNZ 2020-00196-2). Patients with samples, where the health record could not be accessed, general consent was refused, or any other statement refusing research was detected, were excluded. For patients hospitalized more than once with EV infections during the study period, only data from the first admission was included.

### Virus Identification and Quantification

We used the Biofire Filmarray Respiratory Panel PCR assay (bioMérieux) for respiratory viruses in routine diagnostics (ISO 17025 accredited). The panel PCR included EV/RV but could not differentiate between these viruses. The assay was used according to the manufacturer’s instructions.

As mentioned, the left-over material of the nasopharyngeal swabs was stored at −80 °C. From these biobanked EV/RV positive respiratory samples, the RNA was extracted using the QIAamp Viral RNA kit (Qiagen) on the automated QIAcube (Qiagen) robot system, as per the manufacturer’s instructions.

We used the extracted samples in the EV-D68 specific real-time RT-PCR,^22^ to detect and distinguish between the following viruses: EsV-D68, other non-EV-D68 and RV.

### Data Collection and Definitions

We generated an electronic case report form using Epidata to collect specific information about the patients. We reviewed the electronic patient’s health records and performed the data extraction to the electronic case report form without knowing the PCR result.

Hospitalized cases included in this study were admitted via the emergency room or ambulatory care. Outpatients were mostly treated in the emergency room.

The collected data included age (in years), gender, baseline disease, place, and date when the swab was done. Clinical course and outcome data include symptoms, complications, underlying conditions, treatment, respiratory severity and discharge diagnosis, such as the need for hospitalization, ICU stay, hospitalization and ICU duration and mortality during hospitalization. For treatment, we recorded the use of oxygen, steroids and antibiotic therapy.

The Pediatric Respiratory Severity Score (PRESS) is a tool for the initial assessment of respiratory tract infections in children for prediction of severity and hospitalization.^23–26^ It consists of 5 parameters: respiratory rate, wheezing, accessory muscle use, SpO_2_, and feeding difficulties. Each gives 0 or 1 point and based on these it is classified into 3 groups: mild (0–1), moderate (2–3) and severe (4–5).

### Statistical Methods

We described differences between EV-D68, non-EV-D68 and RV-positive children. Data were presented as proportions, or medians with interquartile ranges (IQR). To assess the impact of infection type on the severity of illness, we used an ordinal regression model. Our primary analysis focused on the 3-level pediatric respiratory severity score, estimating the effect of EV-D68 infection compared with non-EV-D68 and RV infections separately. We assumed that the effect of the predictor on the odds of higher severity levels was equal. To test this assumption, given the limited number of cases in our study, we performed 2 separate logistic regressions.

To further evaluate the proportional odds assumption of the ordinal logistic regression, we conducted additional logistic regressions. These models collapsed the severity scores into 2 categories: 1 versus 2 or 3, and 1 and 2 versus 3. Although the estimates exhibited slight variations due to the small sample size, all coefficients consistently indicated the same direction and fell within a similar range. Therefore, we conclude that the results of the ordinal regression analysis are reliable.

In the secondary analysis, we investigated whether children with EV-D68 were more likely to be admitted to the ICU compared with those with non-EV-D68 or RV infections. A logistic regression model was fitted, with virus type as the predictor variable and patient age included as a covariate to account for its potential influence.

The standardized mean difference (SMD) for binary and categorical variables was calculated following Austin^27^ and Yang and Dalton, respectively.^28^ The use of these SMDs for continuous as well as binary and categorical variables allows us to quantify the difference between the means of the outcome variable in the 3 groups, even variables of different measurement levels. SMD values above 0.8 indicate a large effect size, which is likely to be clinically significant. Values between 0.5 and 0.7 indicate a medium effect size, which may be clinically relevant, and >0.2 only a small effect size.^29^

These statistical analyses aimed to provide insights into the association between EV-D68 infection and illness severity as well as ICU admissions among pediatric patients.

## RESULTS

### Patients

**FIGURE 1.**
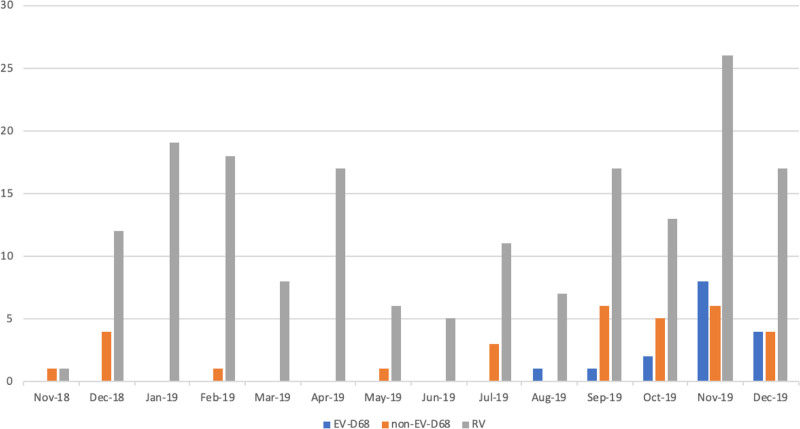
Monthly distribution of EV-D68 infections, Basel November 2018–December 2019.

During the study period from November 2018 to December 2019, we identified a total of 224 cases, of which 16 (7%) were EV-D68, 31 (14%) were non-EV-D68 and 177 (79%) rhinovirus infections. All cases of EV-D68 occurred from August to December 2019, with the majority in November (n = 8; 50%) and December (n = 4, 25%) (Fig. 1).

Patient characteristics are presented in Table 1. There was a slight male predominance in all patients, especially in the EV-D68-positive patients (75% male). The median age in the EV-D68 group was 13 months (range 0 months–13 years). The median age was 3 and 8 months for non-EV-D68 (range 0 months–6 years) and RV-infected patients (range 0 months–19 years), respectively. We observed that patients with EV-D68 had higher percentages of underlying lung disease than those with non-EV-D68/RV (12.5% vs. 3.2% vs. 5.6%).

**TABLE 1.** Demographic Characteristics, Underlying Conditions, and Outcomes

	EV-D68 n = 16	Non-EV-D68 n = 31	RV n = 177	SMD
Age, year—median (IQR)	1.1 [1.03, 3.28]	0.03 [0.0, 1.06]	0.08 [0.01, 2.08]	0.478
Males, no (%)	12 (75)	14 (45)	87 (49)	0.424
Underlying diseases, no. (%)	2 (13)	7 (23)	56 (32)	
Cardiologic diseases	0 (0)	2 (7)	15 (6)	0.315
Prematurity	0 (0)	3 (10)	13 (7)	0.177
Lung diseases	2 (13)	1 (3)	10 (6)	0.218
Oncologic diseases	0 (0)	0 (0)	9 (5)	0.124
Autoimmune diseases	0 (0)	1 (3)	4 (2)	0.236
CNS diseases	0 (0)	0 (0)	3 (2)	0.293
Hematologic diseases	0 (0)	0 (0)	2 (1)	0.101
Outcomes				
Hospitalization, no (%)	16 (100)	27 (87)	163 (92)	0.374
Hospitalization –				
LOS (d)- median (IQR)	2.5 [2.0, 4.5]	2 [1.0, 4.0]	2 [1.0, 3.0]	0.241
ICU – no. (%)	4 (25.0)	6 (19)	10 (6)	0.372
ICU – LOS (d) - median (IQR)	3.5 [2.0, 4.25]	1.5 [1.0, 8.75]	1.0 [1.0, 2.0]	0.834
Death – no. (%)	0 (0)	0 (0)	0 (0)	<0.001
PRESS – no (%)				
Mild	2 (13)	22 (71.0)	117 (66)	-
Moderate	6 (38)	5 (16)	43 (24)	-
Severe	8 (50)	4 (13)	17 (10)	-
Components of PRESS – no (%)				
Tachypnea	11 (69)	9 (29.0)	41 (23)	0.676
Wheezing	10 (63)	6 (19)	28 (16)	0.719
Accessory muscle use	12 (75)	6 (19)	53 (30)	0.867
Oxygen saturation <95%	11 (70)	9 (29)	39 (22)	0.696
Feeding difficulties	1 (6)	4 (13)	52 (29)	0.425

CNS, central nervous system; LOS, length of stay in days.

Percentage or median and interquartile range (IQR) are shown.

### Clinical Presentation

Table 2 shows the most common symptoms at the time of clinical testing, complications and treatment of patients by causative viral infection.

**TABLE 2.** Symptoms, Complications and Treatment

	EV-D68 n = 16	Non-EV-D68 n = 31	RV n = 177	SMD
Symptoms – no. (%)				
Cough	14 (88)	15 (48)	111 (63)	0.604
Wheezing	10 (63)	6 (19)	28 (16)	0.719
Dyspnea	12 (75.0)	7 (23)	40 (23)	0.821
Rhinitis	8 (50.0)	20 (65)	118 (67)	0.228
Sore throat	0 (0.0)	1 (3)	11 (6)	0.255
Hoarseness	0 (0.0)	0 (0.0)	7 (4)	0.191
Nausea	0 (0.0)	1 (3)	4 (2)	0.177
Vomiting	2 (13)	9 (29)	37 (21)	0.277
Diarrhea	0 (0.0)	1 (3)	13 (7)	0.28
Ear ache	1 (6)	1 (3)	12 (7)	0.109
Conjunctivitis	0 (0.0)	2 (7)	8 (5)	0.255
Exanthema	1 (6)	3 (10)	14 (8)	0.085
Fever	6 (38)	10 (32)	67 (38)	0.078
Meningism	0 (0.0)	1 (3)	2 (1)	0.184
Feeding difficulties	1 (6)	4 (13)	52 (29.4)	0.425
Complications – no. (%)	6 (38)	6 (19)	11 (6)	
Neurological	1 (6)	0 (0)	0 (0)	0.243
Pneumonia	3 (19)	0 (0)	6 (3)	0.483
Febrile seizure	1 (6)	3 (10)	1 (1)	0.289
Meningitis/Encephalitis	1 (6)	3 (10)	4 (2)	0.214
Treatment – no. (%)				
Oxygen supplementation	11 (69)	7 (23)	23 (13)	0.892
Antibiotics	9 (56)	11 (36)	62 (35)	0.291
Steroids	8 (50)	5 (16)	26 (15)	0.542

Column SMD (standardized mean difference) contains the average of the standardized mean differences between individual arms.

Patients with EV-D68 presented mostly with cough (88%), wheezing (62%) and dyspnea (75%). Other common symptoms in the non-EV-D68 and RV group were sore throat, vomiting and feeding difficulties. Among all patients, there was no difference in the presence of fever between patients with EV-D68 compared with non-EV-D68 and RV.

In Table 1, the distribution of the respiratory severity scores in the 3 patient groups is shown. It appears that higher severity levels appear more likely with EV-D68 infection. Since it is difficult to test this assumption, with the low number of cases in this study, we performed 2 separate logistic regressions. Accordingly, the odds of having a more severe outcome with EV-D68 infection is increased by a factor of about 11.6 [confidence intervals (CI): 3.51–41.15] relative to EV, and by about 9.9 relative to RV (CI: 3.75–27.95). The 95% CI excludes an odds ratio (OR) of 1, which mirrors the low *P* values (both *P* = 0.000) in our ordinal regression (see Tables, Supplemental Digital Content 1 and 2, http://links.lww.com/INF/F675).

Nineteen percent patients with EV-D68 received a diagnosis of secondary bacterial pneumonia based on various combinations of clinical presentation, laboratory values and radiograph findings compared with only 3% in the RV and 0% in the non-EV-D68 groups, with a SMD of 0.483. Overall, our numbers indicate a moderate, potentially clinically relevant difference toward a higher likelihood for complications in the EV-D68 group compared with the others, including 1 case of possible acute flaccid myelitis. Of note, this was a 2-year-old and 8-month-old boy who was admitted with a 5-day history of fever, rhinitis, cough and refusal to walk without pain in the legs. He had acute flaccid paralysis of the right leg and investigation of the cerebrospinal fluid (CSF) showed pleocytosis (159 leukocytes/mm^3^, of which 42/µl were polynuclear cells, with normal protein and glucose levels). Multiplex PCR of the CSF was positive for human herpesvirus-6. Unfortunately, specific PCR for enteroviruses in CSF was not performed, whereas enterovirus PCR was positive (and diagnosed as EV-D68 in this current study) in a nasopharyngeal specimen. During his disease, paraplegia and loss of strength in the right arm also occurred. His first magnetic resonance imaging showed signs of myelitis. He was treated with Ganciclovir, intravenous immunoglobulins, and a high dose of methylprednisolone. At 3.5 years of age, a follow-up visit revealed persistent paralysis of the right leg and his walking capability was still severely limited whereas the strength of the right arm had much improved.

### Clinical Management and Hospital Course

Most tested patients (212/224, 95%) presented to the emergency room. All 16 patients with EV-D68 required hospitalization, whereas 13% and 8% of the non-EV-D68 and RV groups, respectively, were managed as outpatients. There were no significant differences in hospital length of stay observed between the groups. None of the patients died.

One-fourth (n = 4; 25%) of the EV-D68 patients needed to be treated in the ICU, with an average length of stay of 3.5 days, with an SMD of 0.834, indicating a significant effect size in the severity of illness among EV-D68 patients requiring ICU treatment.

We fitted a logistic regression model predicting ICU stay (yes/no) by virus type, accounting for patients’ age as a covariate. With a *P* value of 0.001, there appears to be an effect such that the likelihood of being admitted to ICU is lower with RV than with EV-D86 infection. There was no significant difference or tendency shown between non-EV-D68 and EV-D68 (*P* = 0.836) The estimated OR showed that the odds of being admitted to the ICU is estimated to be about 5 times more likely with EV-D68 infection compared with RV (OR: 5.4; 95% CI: 1.23–19.23, see Tables, Supplemental Digital Content 3 and 4, http://links.lww.com/INF/F676).

Regarding treatment (Table 1), patients with EV-D68 infections were more likely to receive medical support in the form of supplementary oxygen, antibiotics, and steroids. Supplementary oxygen was the most used treatment in the EV-D68 group, with 69% compared with 36% in the non-EV-D68 and RV groups, with an SMD of 0.892.

## DISCUSSION

This study examined a cohort of 224 pediatric patients with EV-D68 and non-EV-D68 EV or RV infection who presented to the University Children’s Hospital Basel between November 2018 and December 2019. We provide valuable information on the characteristics of EV-D68 respiratory tract infections in children in North-western Switzerland, aiding in healthcare planning for future outbreaks and enhancing our understanding of the disease. Our findings indicate that EV-D68 is more common in children in Switzerland than previously thought. In general, EV-D68 infections were more severe and had higher morbidity, resulting in higher hospitalization rates and ICU admissions as compared with those with non-EV-D68 or RV infections.

There was a male predominance (75%) among EV-D68 patients, consistent with many other respiratory viral infections.^30–32^ Muenchhoff and Goulder^31^ postulated that this can be partially explained by the observation of stronger T-helper 1 cell immune responses in females than in males.

Interestingly, the median age of children presenting with EV-D68 in our study was 21 months, which is similar to many other studies reporting a median age of 24 months or older for EV-D68 infections.^7,33–35^

In agreement with previous studies, wheezing was commonly observed in children with EV-D68 respiratory tract infections.^36–38^ The high proportions of wheezing in EV-D68 patients likely contributed to the increased use of corticosteroid therapy, which is a known targeted treatment for severe cases of lower respiratory tract infections associated with wheezing.

Previous studies have shown that preexisting asthma and recurrent wheezing are associated with medically attended EV-D68 infection.^39,40^ In our cohort, we observed that underlying lung diseases were more common in children with EV-D68 infections than in the other groups, yet with an SMD of 0.236, that is, a small size effect. However, the reliability and validity of early childhood asthma diagnosis can vary across different hospitals and studies, and the inclusion of recurrent or prior wheezing was not consistently accounted for in our study.

Most patients in the EV-D68 group were previously healthy, underscoring the significant disease burden of EV-D68 in previously healthy children. EV-D68 infections were associated with more severe presentations, evidenced by higher rates of wheezing, tachypnea, accessory muscle use and low oxygen saturation, compared with the non-EV-D68 and RV groups. This led to higher PRESS-Scores for EV-D68 patients, with the odds of having a more severe presentation with EV-D68 infection being significantly higher compared with non-EV-D68 and RV infections. The severity of EV-D68 is also demonstrated by the fact that children with EV-D68 infections were 5.4 times more likely to require ICU admissions compared with RV infections.

EV-D68 patients received more corticosteroids, antibiotics and oxygen therapy, likely due to the more severe respiratory symptoms associated with this infection.

Schuster et al^40^ showed similar findings in a retrospective review that EV-D68-infected children were more likely to present with a recurrent wheeze or cough and received significantly more likely respiratory therapies such as albuterol and aminophylline, even those without a history of asthma. The intensity of such therapies often requires an intensive care setting, thereby leading to more ICU admissions.

Savage et al^41^ described a large cohort of 511 pediatric patients with enterovirus/rhinovirus infections and found more severe clinical symptoms in those 170 with EV-D68 infection compared with the rest with non-EV-D68 infections. They used an asthma score (Pediatric Asthma Severity Score) consisting of wheezing, prolongation of expiration and work of breathing. Higher scores were associated with a higher likelihood of hospitalization and the use of albuterol and steroids. Nonetheless, severe complications, prolonged hospitalizations, and death were not more common in EV-D68 respiratory illness, consistent with previous reports. While no fatalities occurred at our institution, increasing evidence links EV-D68 infection with AFM or AFP.^13,42,43^ Bigi et al^44^ from Bern, Switzerland, recently reported the first documented case of AFM in Switzerland. Although our cohort is too small to draw firm conclusions, identifying 1 patient with AFP highlights the importance of further research and monitoring to understand potential complications associated with EV-D68 infection. Therefore, further surveillance of complications associated with enterovirus infections, ideally in multicenter studies, using existing tools such as the Swiss Pediatric Surveillance Unit^45^ or the Swiss Pathogen Surveillance Platform (PMID 37171846) should be performed.

Finally, important limitations of our study include a retrospective chart review and limited historical data, which warrant cautious interpretation of the findings.

In conclusion, EV-D68 has caused significant outbreaks worldwide, primarily presenting with wheezing and severe respiratory infections. The relationship between EV-D68 and asthma severity requires further investigation. Vigilance and continued research are crucial for effectively managing this viral infection and minimizing its impact on pediatric populations.

## ACKNOWLEDGMENTS

*We are grateful to all health care professionals at the University of Basel Children’s Hospital who were involved in the diagnosis and treatment of patients with enterovirus infections included in this study.*

## Supplementary Material


