# Supplementary material for: Clinical Presentation of Enterovirus D68 in a Swiss Pediatric University Center
Source: Pediatr Infect Dis J. 2024 Aug 14;43(12):1135–40. doi: 10.1097/INF.0000000000004503 (PMC11542972; doi:10.1097/INF.0000000000004503)
Supplement: Supplementary file 1 [file inf-43-1135-s001.pdf]

**SUPPLEMENTAL DIGITAL CONTENT 1.** Results of ordinal logistic regression model, to estimate whether the odds of having higher rather than lower severity level depend on the type of infection.

| | Estimate | Std. Error | z value | p value |
|---|---|---|---|---|
| **EV vs EV-D68** | -2.447 | 0.624 | -3.919 | 0.000 |
| **RV vs EV-D69** | -2.295 | 0.508 | -4.521 | 0.000 |

**SUPPLEMENTAL DIGITAL CONTENT 2.** Transformed estimated odds ratios of having a higher severity level under EV-D68 than under EV and RV respectively, and their 95% confidence intervals.

| | **OR** | **2.50%** | **97.50%** |
|---|---|---|---|
| **EV-D68 vs EV** | 11.6 | 3.51 | 41.15 |
| **EV-D68 vs RV** | 9.9 | 3.75 | 27.95 |
