# Supplementary material for: Clinical Presentation of Enterovirus D68 in a Swiss Pediatric University Center
Source: Pediatr Infect Dis J. 2024 Aug 14;43(12):1135–40. doi: 10.1097/INF.0000000000004503 (PMC11542972; doi:10.1097/INF.0000000000004503)
Supplement: Supplementary file 2 [file inf-43-1135-s002.pdf]

SUPPLEMENTAL DIGITAL CONTENT 3. Results of logistic regression model regression regarding ICU admission on infection type and age.

	Estimate	Std. Error	z value	p value
**EV vs EV-D68**	-0.155	0.750	-0.207	0.836
**RV vs EV-D68**	-1.689	0.668	-2.530	0.011
**Age (years)**	0.082	0.060	1.383	0.167

SUPPLEMENTAL DIGITAL CONTENT 4. Estimated odds ratios of begin admitted to the ICU and their 95% confidence intervals.

	**OR**	**2.50%**	**97.50%**
**EV D68 vs EV**	1.168	0.250	5.037
**EV D68 vs RV**	5.416	1.323	19.281
**Age (years)**	1.086	0.951	1.211
